# Limited segmental rectal resection in the treatment of deeply infiltrating rectal endometriosis: 10 years’ experience from a tertiary referral unit

**DOI:** 10.1093/gastro/gou055

**Published:** 2014-08-21

**Authors:** James English, Muhammad S. Sajid, Jenney Lo, Guy Hudelist, Mirza K. Baig, William A. Miles

**Affiliations:** ^1^Department of Obstetrics and Gynaecology, Brighton and Sussex University Hospitals NHS Trust, 177 Preston Road Brighton, BN1 AG, UK, ^2^Department of General, Endoscopic & Laparoscopic Colorectal Surgery, Western Sussex Hospitals NHS Foundation Trust, Worthing Hospital, Worthing, West Sussex, BN11 2DH, UK, ^3^Department of Obstetrics and Gynaecology, Western Sussex Hospitals NHS Trust, Worthing Hospital, Worthing, West Sussex, BN11 2DH, UK and ^4^Department of Obstetrics and Gynaecology, Wilhelminen Hospital, Vienna, Austria

**Keywords:** rectal endometriosis, limited segmental anterior rectal resection, long-term outcomes, quality of life

## Abstract

**Background.** The management of symptomatic rectal endometriosis is a challenging condition that may necessitate limited stripping or limited segmental anterior rectal resection (LSARR) depending upon the extent and severity of the disease.

**Objective.** To report the efficacy of LSARR in terms of pain, quality of life and short- and long-term complications—in particular, those pertaining to bowel function.

**Methods.** The case notes of all patients undergoing LSARR were reviewed. The analysed variables included surgical complications, overall symptomatic improvement rate, dysmenorrhoea, dyspareunia, and dyschezia. Chronic pain was measured using a visual analogue scale. Quality of life was measured using the EQ-5D questionnaire. Bowel symptoms were assessed using the Memorial Sloan Kettering Cancer Centre (MSKCC) questionnaire.

**Results.** Seventy-four women who underwent LSARR by both open and laparoscopic approaches were included in this study. Sixty-nine (93.2%) women reported improvement in pain and the same percentage would recommend the similar procedure to a friend with the same problem. Approximately 42% of women who wished to conceive had at least one baby. The higher frequency of defecation was a problem in the early post-operative period but this settled in later stages without influencing the quality of life score. Post-operative complications were recorded in 14.9% of cases.

**Conclusions.** LSARR for rectal endometriosis is associated with a high degree of symptomatic relief. Pain relief achieved following LSARR does not appear to degrade with time. As anticipated, some rectal symptoms persist in few patients after long-term follow-up but LSARR is nonetheless still associated with a very high degree of patient satisfaction.

## INTRODUCTION

Rectal endometriosis is one of the most technically difficult diseases to treat surgically, since it frequently requires multidisciplinary skills. Up to 10% of women with endometriosis will have rectovaginal disease [1]. Evidence has been building over the past few years, that excision of rectal deeply infiltrating endometriosis (DIE) is associated with faster resolution of symptoms [2–5], but doubts have been expressed about resection-related complications [6–8]. The current literature reports limited information regarding bowel symptomatology following limited segmental anterior rectal resection (LSARR) for endometriosis. Various studies have reported encouraging results following excision of rectal DIE by segmental resection, with very low complication- and recurrence rates [5, 9], but others have recommended long-term follow-up of symptomatology prior to drawing conclusive evidence from short-term outcomes [10]. Studies comparing the quality of life following anterior resection of the rectum for rectal cancer within the general population fail to demonstrate significant difference in the health-related quality of life; however, these patients with rectal cancer did not have long-standing underlying disease [11]. Yet, other authors argue that the radical surgery for endometriosis is unnecessary and recommend shaving of disease from the rectum only, because it results in lower post-operative complication rates [12, 13].

The radical nature of the surgery, together with lack of anatomical basis for LSARR and higher learning curve, can lead to serious bladder dysfunction, rectal dysfunction, and sexual dysfunction following surgery for DIE. Although the ‘classical' laparoscopic LSARR for DIE involving segmental bowel resection has been proven to relieve symptoms successfully, its efficacy has apparently been hampered by several post-operative long-term and/or definitive pelvic dysfunctions, directly or indirectly influencing the health-related quality of life. However the alternative approach, in the form of ‘nerve sparing' LSARR, has been reported with encouraging results [14], but still needs validation by a high powered, randomized, controlled trial. The relationship between DIE of the rectum and functional bowel symptoms, as well as the impact of LSARR on bowel symptoms, appears increasingly complex. With the exception of cases in which the DIE leads to direct rectal stenosis, it seems likely that certain bowel symptoms are a result of cyclic inflammatory phenomena that lead to irritation of the rectum, and not necessarily the result of actual involvement of the rectum by the DIE itself, nor LSARR, because they frequently occur in women free from rectal nodules. Functional or inflammatory bowel diseases and rectal hypersensitivity may be associated with pelvic endometriosis and consequently jeopardize the hypothetical causal relationship between the presence of a rectal nodule and bowel complaints. Women treated by LSARR for rectal endometriosis may continue to experience post-operative bowel complaints, such as constipation, painful defecation, increased frequency of defecation and tenesmus [15].

This article presents the clinical results of women with DIE in the rectum, who underwent LSARR in a tertiary care centre over a ten-year period from 2000 to 2010, and reports in depth on ensuing bowel symptoms. Since we first started to perform this surgery in 2000, it has been our practice to aim for the complete extirpation of all macroscopic disease. Previously published data from our unit has shown that 25% of women with rectovaginal endometriosis require segmental rectal resection in order to achieve macroscopic clearance of the disease whereas, in approximately 70% of women, complete removal of the endometriosis can be achieved by shaving alone [3]. Thus, this article reports that 25% women with the most severe form of DIE required LSARR to ensure its complete clearance.

## PATIENTS AND METHODS

### Inclusion criteria

This was a retrospective study on all patients undergoing LSARR for rectal DIE between 2000 and 2010 at Worthing Hospital. Women who had had previous hormonal therapy for endometriosis requiring LSARR due to symptomatic DIE were also included in the present study.

### Diagnostics

LSARR was performed in women with a single, large, rectal nodule typically at least three centimetres in diameter, those with multiple nodules, and those with features of rectal obstruction, or in patients with extensive DIE over a large surface area of the rectum. The diagnostic pathway for women with suspected endometriosis was adopted from the recommendations published by the European Society of Human Reproduction & Embryology (ESHRE) Guidelines Development Group. Various diagnostic tools used to diagnose symptomatic DIE are (i) laparoscopy to diagnose abdominal, pelvic, rectal and rectal vaginal endometriosis, (ii) transvaginal sonography in the diagnosis of rectal endometriosis, (iii) 3D sonography in the diagnosis of rectovaginal endometriosis, (iv) magnetic resonance imaging in the diagnosis of peritoneal and rectovaginal pouch endometriosis, (v) biomarkers in the diagnosis of endometriosis and (vi) barium enema, endo-anal ultrasound and magnetic resonance imaging of the rectum, to establish the extent of disease.

### Data source and collection

An audit application was submitted to the Audit & Research Department of Western Sussex Hospitals NHS Trust and formal approval was received to conduct this study. Patient case notes were reviewed to record the in-hospital post-surgical outcomes. The questionnaires to assess long-term outcomes were posted to the women by recorded delivery. The questionnaire included a validated psychometric 18-item bowel function scale, known as the ‘Memorial Sloan Kettering Cancer Centre (MSKCC) bowel function instrument', for evaluating bowel function after LSARR, the short-form endometriosis health profile questionnaire (EHP-5) and a global single-item rating of health change (EQ-5D visual analogue score) [16]. Clinical cure was defined as either complete pain relief or significantly reduced pain following LSARR.

Outcome options were (i) surgical complications, (ii) overall symptomatic improvement rate, (iii) dysmenorrhoea, (iv) dyspareunia, (v) dyschezia, (vi) chronic pain pelvic pain, or (vii) variables given in MSKKK, EHP-5 and EQ-5D to access post-operative health-related quality of life.

### Statistical analysis

The collected data were transferred to a Microsoft Excel spreadsheet and analysis was performed using same. Where applicable, the Chi-squared test and Kruskal-Wallis test were used to compare various outcomes. A *P*-value of less than 0.05 was set as significant.

## RESULTS

**Figure 1.**
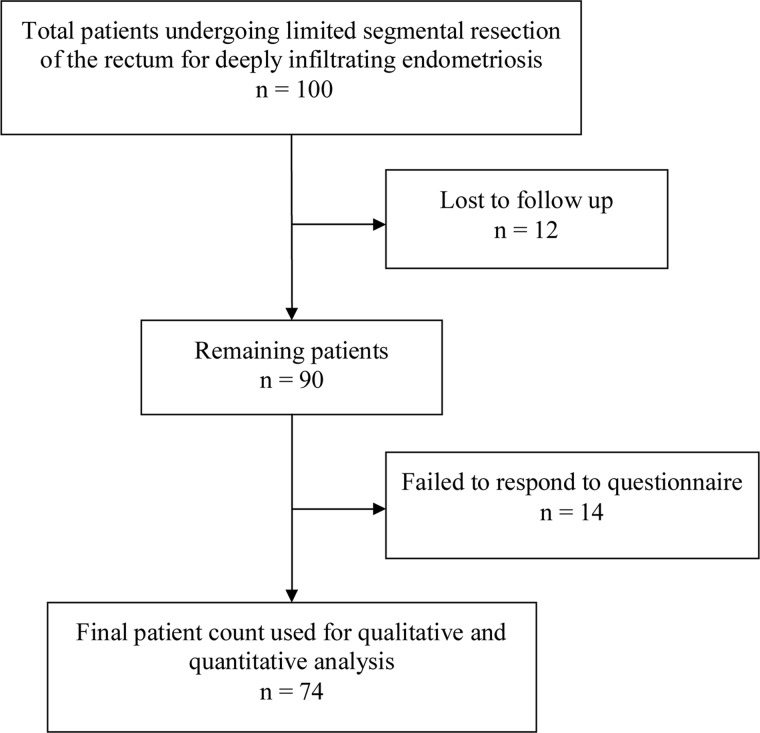
Study flow diagram.

One hundred women underwent LSARR between October 2000 and February 2010. Twelve patients were lost to follow-up due to change in address and were therefore excluded from the final analysis. In addition, 14 patients did not respond to the posted questionnaire (Figure 1). Seventy-five LSARR procedures were performed by an experienced gynaecologist, assisted by a colorectal surgeon with ample experience of anterior resections of the rectum, using a minimally invasive laparoscopic approach. Procedures performed concurrently with LSARR in all patients are shown in Table 1. The operative and in-hospital post-operative complications, experienced by those patients from our cohort who responded to the questionnaire after undergoing LSARR, are shown in Table 2.

**Table 1.** Surgical procedures performed concurrently with LSARR in all patients (*n = *100)

Procedure	Patients (*n*)
Hysterectomy	38
Unilateral oophorectomy	10
Bilateral oophorectomy	11
Defunctioning stoma	24
Sigmoid colectomy	3
Caecectomy/terminal ileal resection	4
Appendectomy	5
Unilateral nephrectomy	2
Partial cystectomy	3

**Table 2.** LSARR-related in-hospital complications in included cohort (*n = *74)

Post-operative complications	Patients *n (*%)
Anastomotic leakage	4 (5.4%)
Fistula	4 (5.4%)
Anastomotic leakage leading to fistula	2 (2.7%)
Ureteric injury requiring JJ stenting	3 (4.1%)
Anastomotic stricture requiring dilation	7 (9.5%)
Total complications	11 (14.9%)

The mean age of the included LSARR patients was 35.8 years (22–49). Sixty-nine women (93.2%) reported reduction in their pain (Table 3). Concurrent hysterectomy doubled the chances of women being completely pain-free (Table 3). The clinical cure rate was 80.8% (42/52) in women who underwent LSARR more than 5 years ago and 77.3% (17/22) in those who underwent LSARR within last 5 years. Of the five women whose pain failed to respond to LSARR, four either failed to conceive or miscarried and the other subsequently underwent a hysterectomy with complete resolution of pain. Histopathological analysis of the resected rectal segment confirmed the presence of DIE in 91.9% patients, fibrosis in 6.8% and combined diverticulosis and fibrosis in 1.4% of those undergoing LSARR. Also, adenomyosis was reported in 54% of women who simultaneously had hysterectomy at the time of LSARR.

**Table 3.** Pain improvement following LSARR in included cohort

Pain	Overall (*n = *74)	Hysterectomy (*n = *32)	No hysterectomy (*n = *42)
Gone	20 (27.0%)	12 (37.5%)	8 (19.0%)
Greatly improved	39 (52.7%)	14 (43.8%)	24 (57.1%)
Somewhat better	10 (13.5%)	5 (15.6%)	6 (14.3%)
No change	2 (2.7%)	0	2 (4.8%)
Worse	3 (4.1%)	1 (3.1%)	2 (4.8%)

**Figure 2.**
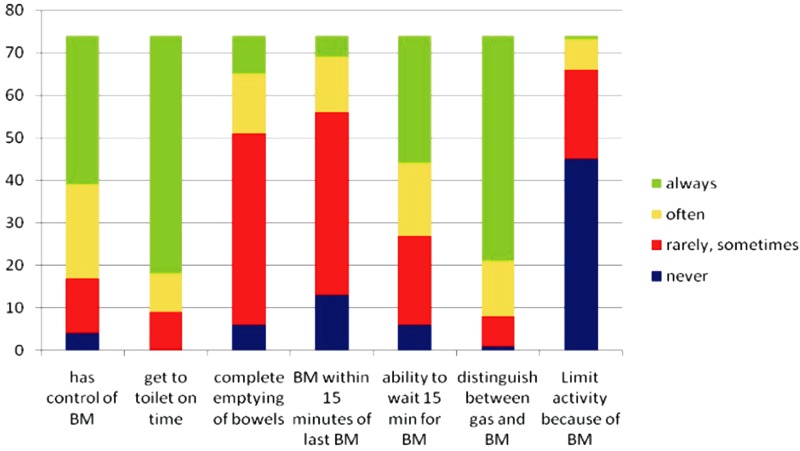
Illustration of results of MSKCC questionnaire (BM = bowel movement).

The effects of LASRR on bowel function, based upon the results of the MSKCC questionnaire, are shown in Figure 2. There was no significant change in control of bowel movement over time (*P* = 0.729; correlation co-efficient, −0.041; Spearman’s non-parametric correlations). Data analysis, using the Independent Samples Kruskal-Wallis test, showed a statistically significant reduction in bowel frequency motions over time, with a mean of 3.7 times daily for women who underwent LSARR in last three years (*P* = 0.031). The mean frequency of defecation dropped to 1.5 per day in women who underwent LSARR approximately 5 years ago. No patient required either bladder evacuation by self-catheterization or assisted bladder evacuation. Of the 74 women who responded, 31 (41.9%) had attempted to conceive: of these 10 (32.3%) went on to have at least one baby. Nine women conceived naturally and five women conceived after *in vitro* fertilization (IVF). There was no significant difference in the age of those who conceived naturally or required IVF (*P* = 0.776; Independent t-test). Sixty-nine women (93.2%) would recommend LSARR to a friend with a similar problem. Of the five women who were unsure or would not recommend the operation, four had failed to conceive or had miscarried.

There was a significant correlation between EHP-5 and time elapsed since LSARR (*P* = 0.037). The mean European Quality of Life 5-Dimensions (EQ5D) Visual Analogue Scale (VAS) scores were consistently lower in our patient group [mean health status score (MHSS) 75.84; SD = 20.26] than in the general population in the south-east of England (MHSS 82.41; SD = 17.30). The greatest difference in the EQ5D VAS score was seen in the 25–34 year age group, with an MHSS of 73.07 in the study population and 85.81 in the general population (*P* < 0.001).

## DISCUSSION

The results of this study are in accord with the results of previously published study cohorts [4, 5, 17] suggesting that the LSARR is highly effective in the treatment of pain associated with rectal DIE. The finding of better quality of life in these patients than in the general population supports previous work from this unit [3], identifies the long-term deleterious effects of severe DIE, as previously highlighted by Garry *et al.* [17], inasmuch as endometriosis of the cul-de sac may result in a diminished quality of life. The overall major complication rate following LSARR for severe DIE has also been previously reported [4, 5, 18–20]. Maytham *et al.* [19] and Boccola *et al.* [20] reported that the segmental resection of the rectum for endometriosis may be associated with a higher complication rate than in surgery for other reasons. Different studies suggest varying anastomotic leakage rates, ranging widely from 0.6–18% [17, 21]. The 5.4% incidence of anastomotic leakage rate in this study cohort was within accepted limits.

Women who underwent an adjunctive hysterectomy were more likely to be completely pain-free. Presumably this accords with the fact that 54% of women who underwent simultaneous hysterectomy were also found to have adenomyosis. Some women had bowel symptoms that did not appear to improve over time; however, the vast majority of these were not severe. Frequency of defecation improved over time and became normal for the vast majority of patients, in line with the findings of Kavalaris [22], and nerve-sparing surgery appears to reduce the need for self-catheterization for bladder evacuation. The reduced pain relief attained by women in this study did not vary with time. This suggests a very low recurrence rate, as previously reported by Dousset *et al.* [5].

Opinion is divided over the appropriate treatment for DIE in the rectum, with some authors favouring limited segmental rectal resection [4, 5, 9], while others recommend the shaving or disk resection of the rectal wall [12, 13]. Yet this debate is only a proxy for the real argument as to whether one should aim for the radical extirpation of all disease or undertake some form of its debulking, commensurate with surgical experience and a desire to limit potential complications. Those who favour the latter approach argue that endometriosis is not cancer and that less-radical surgery is more appropriate. In undertaking LSARR, the surgeon aims to achieve its complete clearance when there is radiological and subsequently histological confirmation of the size and depth of the lesions. The extent of debulking surgery is operator-dependent, sometimes almost total, at times minimal: there is no histology and no precise determination of the extent of residual disease. Thus, studies purporting to demonstrate excellent results through shaving disease from the rectum may be academically invalid, since complete removal of the disease may well have been achieved in the majority of women and there is no accurate estimation of the extent of residual disease when all results are pooled. Thus any comparison between the surgical results achieved by those who perform complete resection for the very worst disease and those who debulk, are intrinsically flawed. Vercellini *et al.* recommended the identification of rectal disease before surgery [23]. Recent reported pre-operative work has supported the validity of transvaginal ultrasonography in the non-surgical diagnosis of rectal DIE [24, 25]. There remains the potential to proceed to a randomized, controlled trial of segmental rectal resection vs. debulking of disease following the ultrasonic identification and quantification of extent of rectal DIE.

The principal argument maintained by colorectal surgeons and gynaecologists who prefer to carry out LSARR in women suffering for DIE, is that this procedure ensures a more complete resection of the rectal disease. This particular hypothesis has been reported quite frequently, comparing LSARR with the removal of rectal nodules [26]. However, microscopically complete resection of rectal implants might remain incomplete even if LASRR is carried out, as indicated by the presence of endometriosis foci found on the margins of resected rectal segment [27–29]. Moreover, the question remains as to whether complete long-term relief from the pain of endometriosis stabilized requires total resection of rectal foci when taking into consideration the risk of post-operative complications and unpleasant functional symptoms. Unlike rectal cancer, rectal endometriosis does not threaten patients' lives; however, it is usually extremely damaging to health-related quality of degree and can influence the regular pattern of bowel activity, resulting in impairment of daily activities. In this article, we chose to focus on both early and long-term post-operative functional outcomes resulting from LSARR employed in the management of rectal DIE, based on the belief that treating endometriosis should not mean reducing pain at the cost of other unpleasant post-operative symptoms. However, this paper does not seek to endorse the superiority of either rectal nodule excision or colorectal segmental resection, because a definitive recommendation must take into account the long-term risk of recurrence associated with each surgical procedure. As the expected recurrence rates of both appear to be closely comparable [30, 31], a comparative study focusing on the risk of recurrences would require several hundred patients, with a follow-up of several years [13]. To our knowledge, no such randomized or prospective comparative study will be available within the next few years. Consequently our study, added to those published by Fanfani *et al.* and Roman *et al.* [30, 31], provides useful information—indispensable when deciding on the most appropriate course of treatment in each individual case of rectal endometriosis—relating to the functional outcomes of surgical procedures such as LSARR.

## CONCLUSION

Radical excision of endometriosis, including LSARR for rectal DIE, is associated with a high degree of symptomatic relief. Pain relief achieved following LSARR does not appear to decrease with time following this procedure. As anticipated, some rectal symptoms persist in few patients over long-term follow-up but LSARR is nonetheless still associated with a very high degree of patient satisfaction.
